# A New Prognostic Algorithm Predicting HCC Recurrence in Patients With Barcelona Clinic Liver Cancer Stage B Who Received PA-TACE

**DOI:** 10.3389/fonc.2021.742630

**Published:** 2021-10-21

**Authors:** Shuyang Hu, Wei Gan, Liang Qiao, Cheng Ye, Demin Wu, Boyi Liao, Xiaoyu Yang, Xiaoqing Jiang

**Affiliations:** ^1^ Eastern Hepatobiliary Surgery Hospital, The Second Military Medical University, Shanghai, China; ^2^ Department of General Surgery, Zhongshan Hospital, Fudan University, Shanghai, China; ^3^ Liver Cancer Institute, Zhongshan Hospital and Shanghai Medical School, Fudan University, Key Laboratory for Carcinogenesis & Cancer Invasion, The Chinese Ministry of Education, Shanghai, China; ^4^ Medical Center of Fudan University, Shanghai, China; ^5^ Department of Health Physical Examination, Shanghai Electric Power Hospital, Shanghai, China

**Keywords:** PA-TACE, hepatocellular carcinoma, nomogram, prognosis, RFS

## Abstract

**Background:**

Postoperative adjuvant transcatheter arterial chemoembolization (PA-TACE) is effective in preventing the recurrence of hepatocellular carcinoma (HCC) in patients treated with surgery. However, there is a lack of reports studying the risk factors associated with recurrence in HCC patients who received PA-TACE. In this study, we identified the independent risk factors for recurrence of HCC patients who received PA-TACE. We also developed a novel, effective, and valid nomogram to predict the individual probability of recurrence, 1, 3, and 5 years after PA-TACE.

**Methods:**

A retrospective study was performed to identify the independent risk factors for recurrence of HCC in a group of 502 patients diagnosed in stage B based on the Barcelona Clinic Liver Cancer (BCLC) evaluation system for HCC that underwent curative resections. Then, subgroup analysis was performed for 184 patients who received PA-TACE, who were included in the training cohort. The other 147 HCC patients were included in a validation cohort. A recurrence-free survival (RFS)-predicting nomogram was constructed, and results were assessed using calibration and decision curves and a time-dependent AUC diagram.

**Results:**

PA-TACE was shown to be a significant independent prognostic value for patients with BCLC stage B [p < 0.001, hazard ratio (HR) = 0.508, 95% CI = 0.375–0.689 for OS, p = 0.002; HR = 0.670, 95%CI = 0.517–0.868 for RFS]. Alpha fetoprotein (AFP), tumor number, tumor size, microvascular invasion (MVI), and differentiation were considered as independent risk factors for RFS in the training cohort, and these were further confirmed in the validation cohort. Next, a nomogram was constructed to predict RFS. The C-index for RFS in the nomogram was 0.721 (95% CI = 0.718–0.724), which was higher than SNACOR, HAP, and CHIP scores (0.587, 0.573, and 0.607, respectively). Calibration and decision curve analyses and a time-dependent AUC diagram were used. Our nomogram showed stronger performance than these other nomograms in both the training and validation cohorts.

**Conclusions:**

HCC patients diagnosed as stage B according to BCLC may benefit from PA-TACE after surgery. The RFS nomogram presented here provides an accurate and reliable prognostic model to monitor recurrence. Patients with a high recurrence score based on the nomogram should receive additional high-end imaging exams and shorter timeframes in between follow-up visits.

## Introduction

Hepatocellular carcinoma (HCC) is one of the most malignant cancers resulting in high morbidity and mortality rates (1, 2). Currently, radical excision and TACE were established and proven to be therapeutic strategies for HCC (3, 4). The Barcelona Clinic Liver Cancer (BCLC) staging system is the most widely used evaluation system to identify therapeutic allocation and prognostic stratification for a patient. However, the presence of heterogeneous characteristics in patients at the same stage, especially in stage B based on BCLC classification has made this difficult (5–8). Furthermore, the recurrence and metastasis rates after resection remain high, and therapy for HCC patients requires more individualized treatment. This is especially for stage B BCLC-classified HCC patients who received (9).

Stage B classified HCC patients comprise a heterogeneous population, and several scoring systems have been proposed to predict the outcomes of TACE in these patients. However, applying these scores to a clinical setting has not been properly validated. Various scoring systems predicting the prognosis of HCC patients receiving different therapies are available. In the setting of TACE, a considerable number of scores, such as Child–Pugh, HAP (10), CHIP (Chiba HCC in intermediate-stage prognostic 2015) (11), and SNACOR (12) aim to predict the prognosis and overall survival of HCC patients undergoing therapy. However, there is a lack of dating when it comes to routine clinical or comparative data between the scores. Therefore, the study presented here aimed to identify the value of PA-TACE in these HCC patients and retrospectively assess the proposed scoring systems in HCC patients eligible for PA-TACE. Moreover, we aimed to identify the predictive factors for survival and construct a novel, individual predicative system for RFS in stage B HCC patients.

## Materials and Methods

### Patients

A group of 502 patients with stage B HCC (based on the BCLC evaluation scale) were retrospectively included in this study. These patients received curative resection surgery at Eastern Hepatobilliary Surgery Hospital, The Second Military Medical University, from 2014 to 2015. The inclusion criteria to include patients in this study were the following (1): patients with precise pathological diagnosis of HCC and assessed at BCLC stage B (2), patients underwent radical resection (3), patients with no complications from other malignant tumors (4), patients with complete clinicopathological and follow-up data, and (5) patients with no evidence of extrahepatic metastasis or primary cancers in other organs. An additional 147 HCC patients who received PA-TACE were included in this study as a validation cohort and contained the same criteria. This study’s protocol was approved by the Clinical Research Ethic Committee.

### Examinations and Follow-Ups

Tumor number and size were measured using enhanced magnetic resonance imaging (MRI) or enhanced computed tomography (CT) before surgery and confirmed during operation. All patients were examined every 3 months during the first 2 years after surgery and every 3–6 months after. Every patient received a routine liver function review, serum alpha fetoprotein (AFP) analysis, chest X-ray, and abdominal ultrasound during follow-up visits. When recurrence was suspected, enhanced CT, enhanced MRI, or positron emission tomography-computer tomography (PET-CT) were used for confirmation.

The period from the time of resection to the time of death or last follow-up was defined as overall survival (OS). RFS was calculated as the period between the operation and time of recurrence. If recurrence was not identified, RFS was calculated from the time of surgery to the time of death or last follow-up.

### Statistical Analysis

Statistical analysis was performed using SPSS version 21 (IBM Corporation, Armonk, NY, USA). Associations between variables were analyzed using the Pearson chi-squared test. Univariate and multivariate analyses of independent prognostic factors were performed using the Cox proportional hazards model. A nomogram was developed using R software version 3.0.2.

## Results

### Patients Demographics and Clinical Characteristics

**Figure 1:**
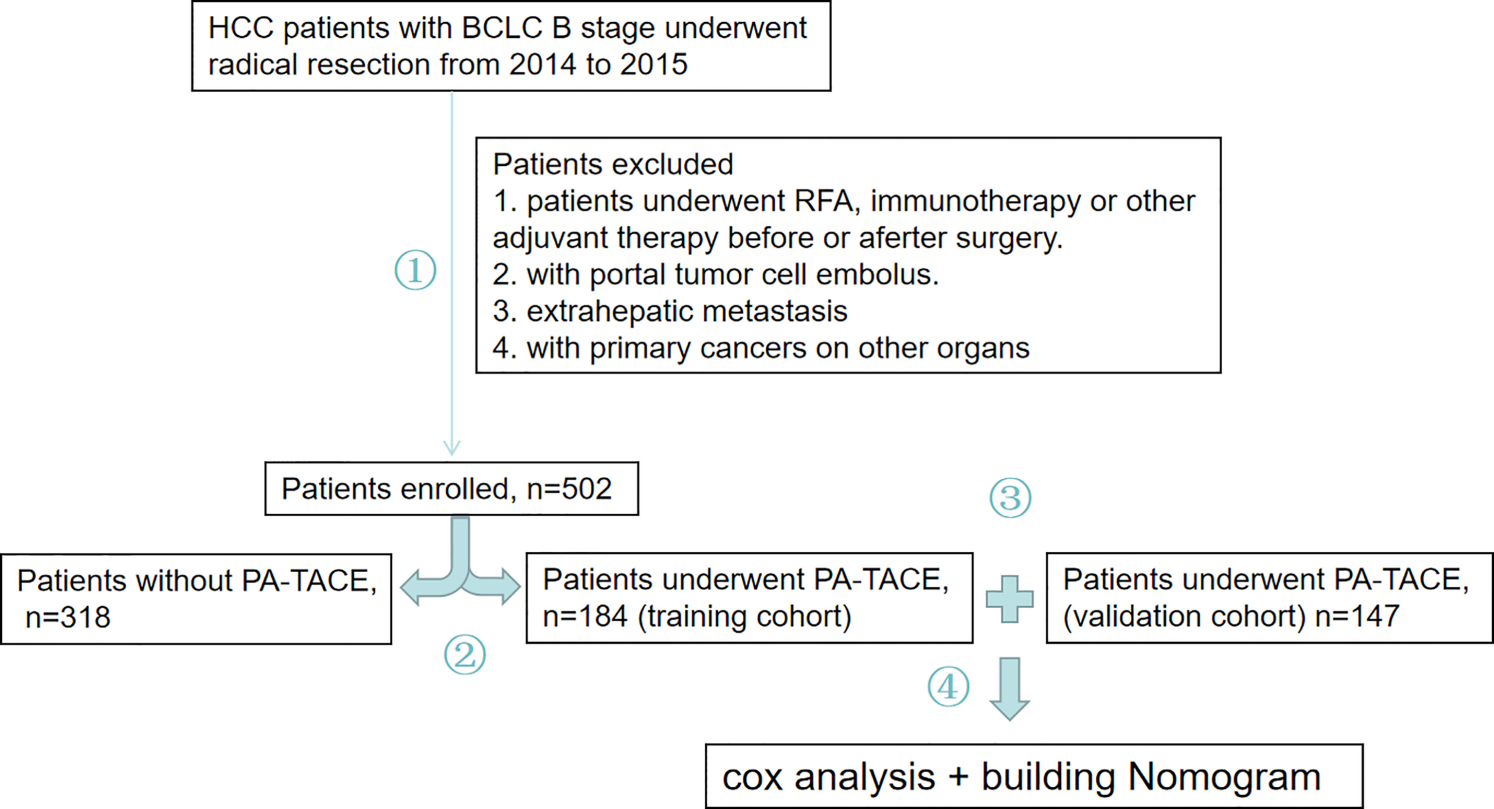
Study overview. 1. Stage B HCC patients who received radical resection from 2014 to 2015 were evaluated. A total of 502 patients were enrolled in our study, and patients were removed based on struct inclusion criteria needed for further analysis. 2. These patients were divided into a PA-TACE group or a group without PA-TACE group. 3. Finally, 184 patients who received PA-TACE were included in the training cohort, and an additional 147 patients who received PA-TACE following the same screening criterion as the training cohort were included in a validation cohort. 4. A novel nomogram was produced to predict RFS in HCC patients who received PA-TACE after radical resection.

The 502 HCC patients were divided into a group that received PA-TACE and a group that did not receive PA-TACE. To construct a reliable and individual predicative system for RFS, 184 patients who received PA-TACE were included in the training cohort, and 147 patients who received PA-TACE following the same screening criterion were included in the validation cohort. After, a novel nomogram was produced to predict RFS for stage B HCC patients who received PA-TACE after radical resection (**Figure 1**).

The detailed clinical characteristics for the patients included in this study are presented in **Table 1**. Most of the patients included in this study were male (83.3%), and 79.1% of the patients included were younger than 60 years of age. In addition, 85.3% of the patients were positive for hepatitis B surface antigen (HBsAg), and 25.9% of the patients were diagnosed with cirrhosis. The clinical characteristics between patients who received or did not receive PA-TACE showed no significant differences (**Table 1**).

**Table 1:** Demographic and clinical characteristics of patients.

Characters	Total patients	P-TACE		
	n = 502	No	Yes	p-value
Gender, male/female	418/84	258/60	160/24	0.092
Age, <60/≥60	397/105	244/74	153/31	0.088
HBsAg,				
negative/positive	74/428	47/271	27/157	0.974
AFP				
<400/≥400 ng/ml	261/241	161/157	100/84	0.422
TBIL				
<20/≥20 µmol/L	432/70	287/34	148/36	0.006
GGT, <45/≥45 U/L	264/238	170/148	94/90	0.608
ALT, <50/≥50 U/L	351/151	225/93	126/58	0.592
ALB, <35/≥35 g/L	31/471	23/295	8/176	0.196
Cirrhosis, no/yes	372/130	228/90	144/40	0.106
Tumor number				
≤3/>3	297/205	278/154	40/30	0.246
Tumor capsule				
No/yes	338/264	152/166	86/98	0.819
Tumor size				
<5/≥5cm	380/222	179/101	139/83	0.761
MVI, no/yes	302/200	189/129	113/71	0.663
Differentiation				
I–II/III–IV	343/159	219/99	124/60	0.732
P-TACE, no/yes	318/184			

APF, alpha fetal protein; MVI, microvascular invasion; TACE, transhepatic arterial chemotherapy and embolization; P-TACE, postoperative TACE.

### Identification of Risk and Protective Factors for OS and RFS in 502 HCC Patients With BCLC Stage B

The results from the univariate analysis found the following associations with OS and RFS, respectively: HBsAg (p = 0.045 and 0.016), AFP (p = 0.001 and 0.027), GGT (p = 0.007 and 0.033), cirrhosis (p = 0.003 and 0.002), tumor number (p < 0.001), tumor size (p < 0.001 and 0.003), MVI (p = 0.001 and < 0.001), differentiation (p = 0.0045 and 0.03), and PA-TACE (p < 0.001 and 0.001). Age (p = 0.02) was also found to have a connection with RFS, and ALB (p = 0.033) was found to be connected to OS. However, based on the multivariate analysis, only tumor number [p = 0.001, hazard ratio (HR) = 1.731, 95% CI = 1.271–2.537], tumor size (p < 0.001, HR = 1.748, 95% CI = 1.304–2.342), MVI (p = 0.023, HR = 1.373, 95% CI = 1.044-1.807), differentiation (p = 0.037, HR = 1.349, 95% CI = 1.018–1.787), and PA-TACE (p < 0.001, HR = 0.508, 95% CI = 0.375–0.689) remained as significant predictors for OS. In terms of RFS, tumor number (p = 0.011, HR = 1.467, 95% CI = 1.093–1.968), tumor size (p = 0.04, HR = 1.313, 95% CI = 1.013–1.703), MVI (p = 0.005, HR = 1.436, 95% CI = 1.118–1.843), PA-TACE (p = 0.002, HR = 0.670, 95% CI = 0.517–0.868) were shown to be influential factors (**Table 2**). Kaplan–Meier survival curves revealed that PA-TACE was a significant prognostic value for both OS and RFS (**Supplementary Figure 1**).

**Table 2:** Univariate and multivariate analyses for OS and RFS in patients with 502 HCC.

Characters	Total patients	Univariate		Multivariate			
		OS	RFS	OS		RFS	
	n = 502	p-value	p-value	p-value	HR (95% CI)	p-value	HR (95% CI)
Gender, male/female	418/84	0.153	0.795	NA		NA	
Age, <60/≥60	397/105	0.134	0.02	NA		NS	
HBsAg,							
Negative/positive	74/428	0.045	0.016	NS		NS	
AFP							
<400/≥400 ng/ml	261/241	0.001	0.027	NS		NS	
TBIL							
<20/≥20 µmol/L	432/70	0.696	0.63	NA		NA	
GGT, <45/≥45 U/L	264/238	0.007	0.033	NS		NS	
ALT, <50/≥50 U/L	351/151	0.128	0.582	NA		NA	
ALB, <35/≥35g/L	31/471	0.033	0.127	NS		NA	
Cirrhosis, no/yes	372/130	0.003	0.002	NS		NS	
Tumor number							
≤3/>3	297/205	<0.001	<0.001	0.001	1.731 (1.271–2.357)	0.011	1.467 (1.093–1.968)
Tumor capsule							
No/yes	338/264	0.191	0.34	NA		NA	
Tumor size							
<5/≥5cm	380/222	<0.001	0.003	<0.001	1.748 (1.304–2.342)	0.04	1.313 (1.013–1.703)
MVI, no/yes	302/200	0.001	<0.001	0.023	1.373 (1.044–1.807)	0.005	1.436 (1.118–1.843)
Differentiation							
I–II/III–IV	343/159	0.045	0.03	0.037	1.349 (1.018–1.787)	NS	
P-TACE, no/yes	318/184	<0.001	0.001	<0.001	0.508 (0.375–0.689)	0.002	0.670 (0.517–0.868)

APF, alpha fetal protein; MVI, microvascular invasion; TACE, Transhepatic arterial chemotherapy and embolization; P-TACE, postoperative TACE; HR, hazard ratio; CI, confidence interval; NA, non analysis; NS, non significant.

### Further Identification of Risk Factors for RFS in the Subgroup Analysis of 184 HCC Patients Who Underwent PA-TACE as the Training Cohort and Another 147 Patients as Validation Cohort

Univariate analysis revealed that age (p = 0.039), AFP (p = 0.008), GGT (p = 0.024), tumor number (p = 0.004), MVI (p = 0.001), and differentiation (p = 0.048) were associated with RFS. Based on multivariate analysis, AFP (p = 0.024, HR = 1.689, 95% CI = 1.072–2.661), tumor number (p = 0.002, HR = 2.021, 95% CI = 1.301–3.138), tumor size (p = 0.028, HR = 1.627, 95% CI = 1.027–2.576), MVI (p = 0.013, HR = 1.751, 95% CI = 1.127–2.721), and differentiation (p = 0.028, HR = 1.657, 95% CI = 1.055–2.600) showed a strong connection to RFS in the training cohort. Surprisingly, AFP, MVI, differentiation, tumor size, and tumor number were found to be significant independent risk values for RFS in the validation cohort (**Table 3**).

**Table 3:** Univariate and multivariate analyses of RFS in training and validation cohort HCC patients underwent PA-TACE.

Characters	Training cohort RFS				Validation cohort RFS			
	n = 184	Univariate	Multivariate		n = 147	Univariate	Multivariate	
		p-value	p-value	HR (95%CI)		p-value	p-value	HR (95% CI)
**Gender, male/female**	160/24	0.903	NA		60/87	0.181	NA	
**Age, <60/≥60**	151/33	0.039	NS		99/48	0.502	NA	
**HBsAg**								
**Negative/positive**	27/157	0.138	NA		16/131	0.983	NA	
**AFP**								
**<400/≥400 ng/ml**	102/82	0.008	0.024	1.689 1.072–2.661)	57/90	0.035	0.031	1.667 (1.048–2.652)
**TBIL**								
**<20/≥20 µmol/L**	148/36	0.868	NA		119/28	0.121	NA	
**GGT, <45/≥45 U/L**	94/90	0.024	NS		79/68	0.032	NS	
**ALT, <50/≥50 U/L**	126/58	0.895	NA		111/36	0.314	NA	
**ALB, <35/≥35g/L**	8/176	0.076	NA		14/133	0.909	NA	
**Cirrhosis, no/yes**	144/40	0.259	NA		95/52	0.045	NS	
**Tumor number**								
**≤3/>3**	112/72	0.004	0.002	2.021 (1.301–3.138)	110/37	0.001	0.007	1.883 (1.185–2.995)
**Tumor capsule**								
**No/yes**	86/98	0.166	NA		43/147	0.842	NA	
**Tumor size**								
**<5/≥5 cm**	100/84	0.011	0.028	1.627 (1.027–2.576)	69/78	0.002	0.036	1.639 (1.033–2.599)
**MVI, no/yes**	113/71	0.001	0.013	1.751 (1.127–2.721)	66/81	0.014	0.04	1.641 (1.023–2.631)
**Times of PA-TACE**								
**<3/≥3**	82/102	0.733	NA		67/80	0.973	NA	
**Differentiation**								
**I–II/III–IV**	124/60	0.048	0.028	1.657 (1.055–2.600)	82/65	0.011	0.028	1.634 (1.054–2.534)

NA, non analysis; NS, non significant.

### A Prognostic Nomogram for RFS and Calibration Curve Evaluation

**Figure 2:**
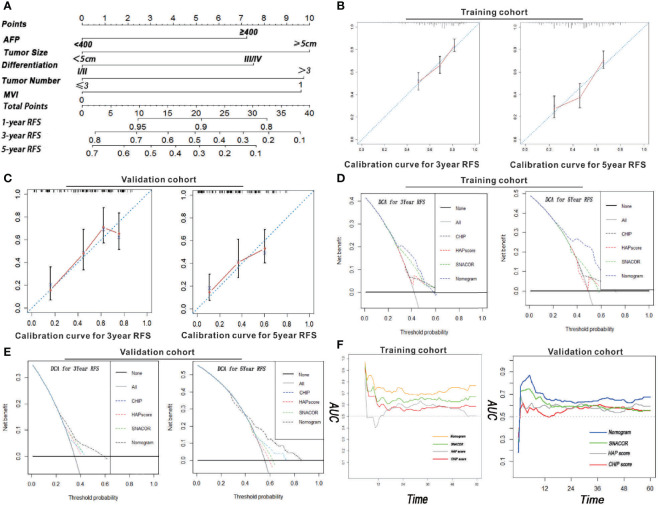
Nomogram, calibration curve, decision curve, and time-dependent AUC analyses for HCC patients who received PA-TACE. **(A)** The novel nomogram constructed to predict RFS was generated by incorporating the variables of AFP, MVI, tumor number, tumor size, and differentiation. The 3- and 5-year calibration curves for predicting RFS in patients in both the training and validation cohorts are illustrated in Panels **(B, C)**. The DCA for the nomogram and common clinical staging systems were used to predict the clinical net benefit in comparison to the integrated nomogram, CHIP, HAP, and SNACOR scores in terms of 3- and 5-year RFS in both the training **(D)** and validation cohorts **(E)**. **(F)** A time-dependent AUC curve for our nomogram compared to CHIP, HAP, and SNACOR. Compared to the other systems, our nomogram showed the greatest prediction for recurrence in both training and validation cohorts.

All independent factors for RFS were integrated into the newly established nomogram (**Figure 2A**). In addition, there was high consistency between the predictions of the nomogram and actual observations as shown based on the calibration curve for RFS both 3 and 5 years after surgery (**Figures 2B, C** **)**.

### Analysis of the New Nomogram Using Both Training and Validation Cohorts

The distinction between the scores obtained for SNACOR, HAP, and CHIP score were compared to determine whether our nomogram was both an efficient and reasonable prognostic model. Even though SNACOR, HAP, and CHIP scores are commonly used for prognosis determination in first-time HCC patients receiving PA-TACE, they do not evaluate RFS. The C-index is a representative method that assesses the degree of consistency between the prediction and actual observations. The distinction ability of our nomogram ranked first based on a C-index of 0.721 (95% CI = 0.718–0.724) and 0.702 (95% CI = 0.699–0.705) for both training and validation cohorts, respectively. These scores were greater than the values obtained using SNACOR, HAP, and CHIP scores (**Table 4**). These results indicate that our new nomogram is a reliable predictor of RFS for HCC patients who receive PA-TACE for the first time.

**Table 4:** Ranking of clinical staging system using C-index for RFS in training and validation cohort.

Variables	Training cohort		Validation cohort	
	c-index	95% CI	c-index	95% CI
Nomogram	0.702	0.699–0.705	0.721	0.718–0.724
AFP	0.571	0.568–0.574	0.563	0.560–0.566
Tumor size	0.563	0.560–0.566	0.573	0.570–0.576
Differentiation	0.544	0.541–0.547	0.553	0.550–0.556
Tumor number	0.587	0.584–0.590	0.612	0.609–0.615
MVI	0.589	0.586–0.592	0.596	0.593–0.599
CHIP	0.582	0.579–0.585	0.607	0.604–0.610
HAP	0.564	0.561–0.567	0.573	0.570–0.576
SNA	0.613	0.610–0.616	0.587	0.584–0.590

### Evaluation of the Clinical Benefit of our New Nomogram Based on Decision Curve Analysis

Decision curve analysis (DCA) is an approach used to estimate the clinical effects of a diagnostic test while considering the subjective nature of risk. In this study, we evaluated the clinical application of our nomogram. These results revealed that the newly constructed tool presented a better net benefit with a higher threshold probability and improved performance for predicting 3- and 5-year RFS than SNACOR, HAP, and CHIP scores in both training and validation cohorts (**Figures 2D** **, E**). Moreover, the AUC diagram also indicated that our nomogram showed stronger overall performance compared with SNACOR, HAP, and CHIP (**Figure 2F**).

## Discussion

Postoperative tumor recurrence greatly threatens the survival of patients diagnosed with HCC (13). PA-TACE is regarded as one of the major treatment measures for HCC patients after surgery. However, there is a lack of reports investigating risk factors for tumor recurrence for HCC patients who underwent TACE treatment after operation (14, 15). In this study, we identified the independent risk factors for recurrence of HCC in patients who underwent curative resection and developed a novel, effective, and valid nomogram for predicting the individual probability of recurrence 1, 3, and 5 years after PA-TACE treatment. AFP, tumor size, tumor differentiation, tumor number, and MVI were included into the nomogram. Moreover, the nomogram presented a high discriminatory ability. In a subsequent study, we used calibration and decision curve analyses to assess the precision of predictions and clinical utility of the nomogram. Our model showed a better net benefit and higher uniformity between the nomogram prediction and the actual observation in both training and validation cohorts.

Many studies have presented the essential role of tumor grade, tumor burden, and liver function in the prognosis of HCC patients before they receive their first TACE therapy (16). The CHIP score, first proposed by Ogasawara et al. in 2015, includes liver function (Child–Pugh score) and tumor characteristics (number, HCV) (11). However, the evaluation of tumor characteristics based only on these two parameters limited the use of this score system in different types of HCC patients. The HAP score takes into account four parameters (ALB, TBIL, AFP, and tumor size) and is divided into four levels (HAP-A, HAP-B, HAP-C, and HAP-D) (10). However, another multicenter research study demonstrated that the TBIL parameter in this tool was meaningless (17). Moreover, the HAP score excludes tumor number, which plays an essential role in CLIP and BCLC (18). The SNACOR score system, first introduced by Kim et al. in 2016, includes liver function, tumor characteristics, and tumor imaging response (12). However, among the subjects included in that study, 32% (109/340) of the patients achieved complete remission based on tumor imaging response after the first TACE treatment. These data are too optimistic, one-sided, and do not match what is observed in actual clinical practice. The ART (19)and ABCR score (20) systems were also two important prediction systems used for HCC patients who were retreated with TACE, allowing for the understanding of the reaction of the tumor from the original treatment. In our study, it is difficult to accurately score patients who may have achieved a tumor-free status when receiving PA-TACE. Hence, the ART and ABCR score systems were not utilized in our study. Moreover, neither scoring system was established for the prediction of RFS in postoperative HCC patients.

A nomogram can be composed of several individual clinical variables and provide personalization for each patient. Based on the data presented in this study, it is apparent that our model is advantageous in the accuracy of predicting prognosis of HCC patients relative to conventional scoring systems (10, 11, 18). Similar to previous work, our results demonstrated that our new nomogram showed greater prediction accuracy for RFS than CHIP (0.607), HAP (0.573), and SNACOR (0.587), having a c-index of 0.721. In a subsequent experiment, our nomogram was retested using calibration and decision curve analyses and showed increasing accuracy and better net benefit for RFS prediction.

Our final nomogram integrated five independent risk factors for RFS of stage B HCC patients who received PA-TACE, including AFP, tumor size, tumor differentiation, tumor number, and MVI. Interestingly, the number of times a patient received postoperative TACE may be a significant risk variate for HCC recurrence. However, this was not considered in our study since most patients who underwent prophylactic treatment usually received PA-TACE two or three times, which does not show a significant difference. AFP is a critical risk factor for recurrence of HCC in patients (21). Tumor size, number, and differentiation are strongly associated with tumor development and metastasis (22–25). MVI is a well-known potential risk factor related to HCC recurrence (26). Early recurrence observed in HCC patients is typically a result of MVI, particularly in regions with tumor thrombus (27–29). Our nomogram displayed better predicative ability for the recurrence of HCC in patients who received TACE. Therefore, our new nomogram can be used to guide routine follow-ups in patients. AFP, tumor size, tumor differentiation, tumor number, and MVI should be observed in patients who received TACE. Moreover, patients who show a high recurrence score as predicted by the nomogram should receive additional high-end imaging examinations, such as MRI or CT exams, and more examinations in a shorter timeframe, even if the last exam after TACE showed no signs of recurrence.

Even though our nomogram works well, there are several limitations that still need to be addressed. First, the study presented here was a retrospective study performed at a single medical center, and additional studies are needed at other centers that verify these findings through prospective studies. Second, stage B HCC patients show great heterogeneity in their characteristics, and thus, markers or identifiers need to be uncovered to evaluate patients who received PA-TACE more precisely. Third, patient selection bias is another factor limiting this study. Finally, the parameter selection system used is subjected to shortcomings and may not fully evaluate all potential parameters.

## Conclusions

In conclusion, stage B HCC patients may benefit from PA-TACE after radical surgery. The RFS nomogram presented in this study provides an accurate and reliable prognostic model for HCC patients classified as stage B based on BCLC who received PA-TACE to facilitate recurrence surveillance. Patients who show a high recurrence score based on the nomogram should receive additional examinations and procedures to closely monitor chances of recurrence.

## Data Availability Statement

The original contributions presented in the study are included in the article/supplementary material. Further inquiries can be directed to the corresponding authors.

## Ethics Statement

Written informed consent was obtained from the individual(s) for the publication of any potentially identifiable images or data included in this article.

## Author Contributions

Study concept and design: XJ and XY. Acquisition of data: SH, LQ, and BL. Analysis and interpretation of data: WG, CY, and SH. Drafting and editing of the manuscript: SH, WG, LQ, and DW. All authors contributed to the article and approved the submitted version.

## Funding

This work was supported by the Chinese Postdoctoral Science Foundation (grant number 2020M671002).

## Conflict of Interest

The authors declare that the research was conducted in the absence of any commercial or financial relationships that could be construed as a potential conflict of interest.

## Publisher’s Note

All claims expressed in this article are solely those of the authors and do not necessarily represent those of their affiliated organizations, or those of the publisher, the editors and the reviewers. Any product that may be evaluated in this article, or claim that may be made by its manufacturer, is not guaranteed or endorsed by the publisher.

## Supplementary Material

Supplementary Figure 1 Kaplan–Meier survival curves for HCC patients who received PA-TACE. (A) RFS curves and (B) OS curves for HCC patients. HCC patients with BCLCclassifiedstage B who received PA-TACE showed a significantly better OS and RFS.
