# Supplementary figures and images for: A New Prognostic Algorithm Predicting HCC Recurrence in Patients With Barcelona Clinic Liver Cancer Stage B Who Received PA-TACE
Source: Front Oncol. 2021 Oct 21;11:742630. doi: 10.3389/fonc.2021.742630 (PMC8566809; doi:10.3389/fonc.2021.742630)

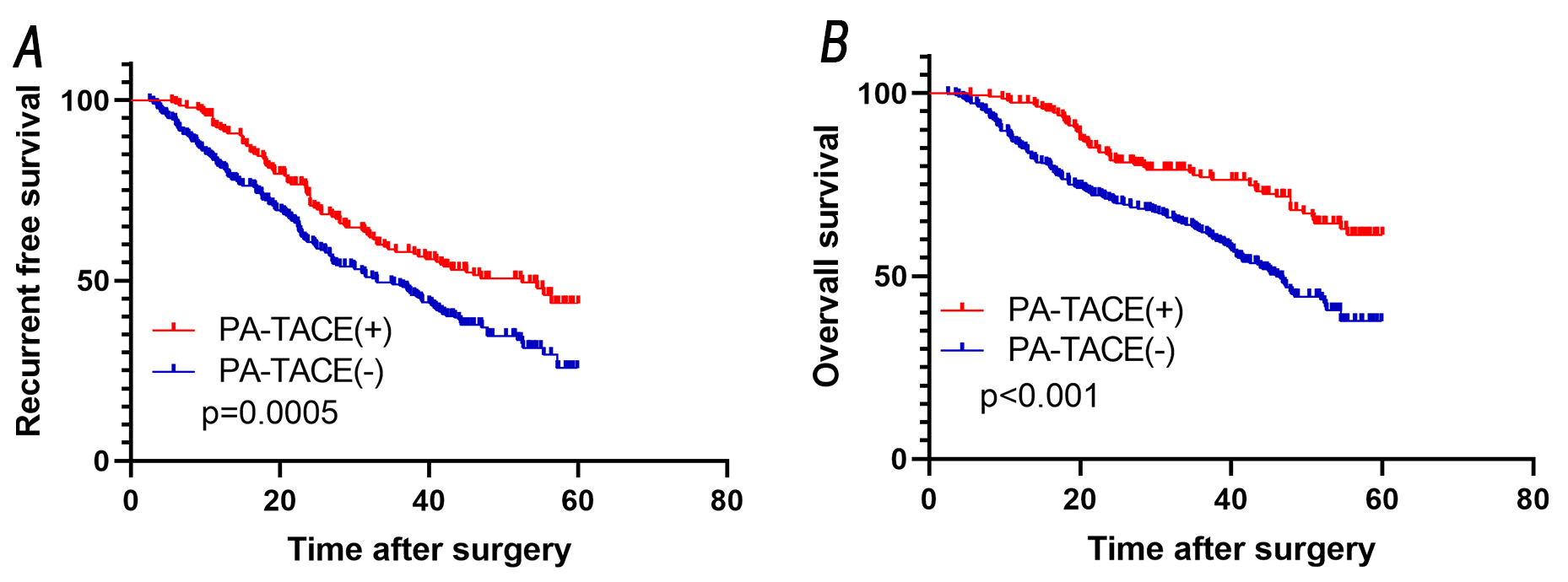

Supplement: Supplementary Figure 1 — Kaplan–Meier survival curves for HCC patients who received PA-TACE. (A) RFS curves and (B) OS curves for HCC patients. HCC patients with BCLCclassifiedstage B who received PA-TACE showed a significantly better OS and RFS. [file Image_1.tif]
